# Policy Narratives on Palliative Care in Sweden 1974–2018

**DOI:** 10.1007/s10728-022-00449-1

**Published:** 2023-01-18

**Authors:** Axel Ågren, Barbro Krevers, Elisabet Cedersund, Ann-Charlotte Nedlund

**Affiliations:** 1 Department of Health, Medicine and Caring Sciences (HMV), Linköping University, Linköping, Sweden; 2 Department of Culture and Society (IKOS), Linköping University, Linköping, Sweden

**Keywords:** Palliative care, Policy narratives, Death denial, Hospice care philosophy, Institutionalised death and dying

## Abstract

In Sweden, efforts to govern end-of-life care through policies have been ongoing since the 1970s. The aim of this study is to analyse how policy narratives on palliative care in Sweden have been formulated and have changed over time since the 1970s up to 2018. We have analysed 65 different policy-documents. After having analysed the empirical material, three policy episodes were identified. In *Episode 1*, focus was on the need for norms, standards and a psychological end-of-life care with the main goal of solving the alleged deficiencies within end-of-life care in hospital settings. *Episode 2* was characterised by an emphasis on prioritising end-of-life care and dying at home, and on the fact that the hospice care philosophy should serve as inspiration. In *Episode 3,* the need for a palliative care philosophy that transcended all palliative care and the importance of systematic follow-ups and indicators was endorsed*.* Furthermore, human value and freedom of choice were emphasised. In conclusion, the increase of policy-documents produced by the welfare-state illustrate that death and dying have become matters of public concern and responsibility. Furthermore, significant shifts in policy narratives display how notions of good palliative care change, which in turn may affect both the practice and the content of care at the end of life.

## Introduction

This article focuses on knowledge governance and explores how the governing instruments of policy-documents can be understood as policy narratives with shifting stories on how palliative care in Sweden should be managed and organised. Over the past decades, there has been an increase in literature, public debates, policy-documents and research on how to raise awareness, end death denial and improve terminal care and cancer care. These efforts have often stemmed from a critique of how death and dying, in Western societies, have been subject to institutionalisation and medicalisation due to relocations of place of death from the home to hospitals and institutions [9, 27]. The development and international success of the hospice movement, with its emphasis on ‘total pain’ acknowledging social, emotional, physical and spiritual needs of the dying, had an important role for the development of palliative care [5, 6]. James and Field [12] argue that the single mindedness and clear definition of problems in the hospice movement have been part of its success. It is, however, argued that this social movement has undergone shifts from opposing acute care of the dying and orthodox cancer care, to being incorporated in the very same field, and thereafter being subject to increased routinisation, bureaucratisation and medicalisation [12]. The expansion of palliative care to general healthcare has led to this type of care being located in the nexus between medical expertise, with a focus on technology and symptom relief, on the one hand, and holistic perspectives originating from the philosophies of the hospice movement on the other [2, 10]. Voices have recently been raised, encouraging a broader public health perspective on palliative care, acknowledging the social contexts and communities of palliative care, and not solely focus on symptoms and health services [1]. Several studies have found that a good death and acceptance of dying has served as a foundation of the hospice care philosophy historically and in palliative care today [14, 15, 26]. A key characteristic of good death, or rather dying well, in late modern societies includes notions of enabling autonomy and dignity of dying persons, although these are represented in limited ways [21]. Making choices at the end of life as a means of maintaining control over one´s life is encouraged within palliative care. Yet, doctors who have expert knowledge, has the ability to offer treatments and deciding on what choices are acceptable or not [25].

Over the last decades, the number of policy-documents has escalated and the term knowledge governance has had a great impact on how matters regarding health and social care should be governed. In many countries where health care is publicly funded, including Sweden, there has been a rise in so-called evidence-based medicine, which has also affected palliative care where calls have been made for palliative care to be universal, more evidence-based and integrated in broader healthcare contexts [19, 22]. However, as argued by several researchers [16, 24], this process of sorting out what constitutes best evidence, deciding on it and implementing what has been decided is tempting but is also oversimplistic. Policy-documents and their inherent policy narratives [18, 20] are powerful governing instruments, that hold interplay of different rationales that each simplifies the phenomenon they are seeking to govern [17], and that further send signals to both the public and care professionals on how issues of concern should be handled and how matters are valued. However, little is known on how such developments affect and construct policies on palliative care in an age when efforts are being made to achieve universal palliative care. The overall aim of this article is to analyse how policy narratives on palliative care in Sweden have been formulated and how they have developed over time from the 1970s up to 2018.

## Methods and Material

In order to understand the processes whereby policies on palliative care have developed and shifted over time in Sweden, we view these types of documents through the theoretical and methodological lens of policy narratives [18]. The point of departure within this theoretical perspective is that various issues are constructed, through policies, as stories with the intention of gaining public acceptance by addressing problems as facts with causal explanations regarding the cause and solution [20]. The process of analysis was, nevertheless, inductive as we had an open approach towards the data and were guided by what we found in the policy-documents analysed, rather than testing an existing theory [4]. The empirical material for this study comprises government investigations, steering documents, care programmes and knowledge support documents^1^ all of which are from Sweden. The Libris database, which stores all publications from the collections of Swedish university libraries, was first used to orient us in the field of end-of-life care in Sweden and finding reports not published by the Swedish Government [41]. The Infotorg database was used to collect material from the Swedish Government [40]. The timeframe of the study was 1974–2018. According to our readings, 1974 was the year when the first policy-document on end-of-life care was published in Sweden. Searches in databases were conducted from January to March of 2019 by author 1 (AÅ) and author 4 (AN). Search terms used was *vård i livets slutskede* (end-of-life care in English) and *palliativ vård* (palliative care), which resulted in 586 documents. The inclusion criteria was that documents should focus on end-of-life care. Consequently, documents where end-of-life care was only mentioned briefly or in the passing, but main focus was on other issues were excluded. These criteria resulted in 65 documents which were downloaded and thus constituted our empirical material. The documents were: government investigations (14); governmental propositions (7); government committee reports (27); government directives (2); reports from the National Board of Health and Welfare (12); and reports from the Regional Cancer Centre (2) and the Swedish Association of Mental Health (1).

## Process of Analysis

The documents were carefully read to gain an overall understanding of the material. After this initial reading, policy narratives were searched for with the purpose of identifying whether the narratives could resemble a story [18]. We identified recurring phrases, terms and expressions and how these occurred, reoccurred and diminished over time. In this process, we were also interested in the way in which narratives conflict with each other, e.g. whether they were polyphonic, contained stories from multiple stakeholders and/or univocal in giving a singular perception of a social problem. Following this approach, we were able to identify three episodes with distinct policy narratives. The division into episodes was based on how narratives were formulated regarding types of phrases, concepts and expressions used. Transitions from one narrative to another occurred when we found significant changes in phrases, concepts and expressions used to give meaning to palliative care, and to describe current problems and solutions within palliative care. The analysis was developed, discussed and conducted by all authors (A1–A4).

## Findings

**Fig. 1:**
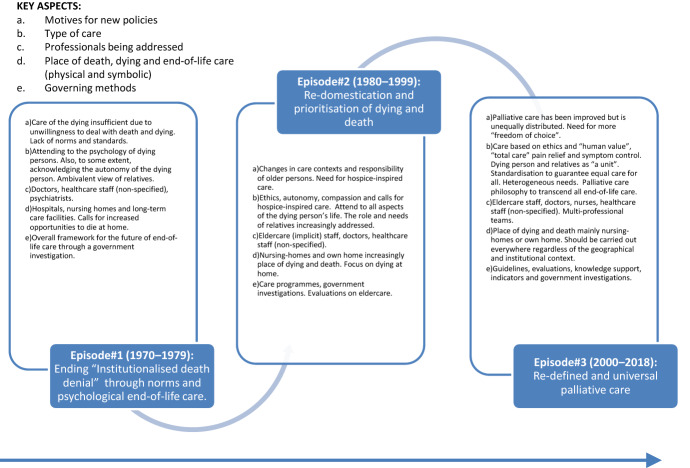
Overview of policy narratives during specific time episodes

In the following section the three policy-episodes identified will be presented. See also Fig. 1 for an overview of main characteristics of the episodes.

### Episode 1: 1974–1979. Ending Institutionalised Death Denial through Norms and Psychological End-of-Life Care

During this episode, it was claimed that death and dying were issues that society and the healthcare sector were not paying attention to and that facing death evoked feelings of insecurity, loneliness and fear. Furthermore, it was said, the development of medical technology resulted in a lack of compassion and empathy from healthcare professionals. When the authors of a governmental report wrote about the historical background on death and dying in Swedish society, the narrative presented argued that *we try to push away thoughts of death* and that *we do not confront death in our lives*. Meanwhile, it was stated, we are constantly surrounded by death in the news and entertainment [36, pp. 63–64]. In a report from the Swedish Association for Mental Health (SAMH) [39], the unsatisfactory ways in which death and dying were managed in healthcare was related to the physical environment of hospitals, as illustrated in *excerpt 1* below:

Death has now been transformed from an often-occurring event in the home with everyone gathered around the deathbed to becoming institutionalised. […] Nowadays, death takes place in silence and in loneliness, in impersonal and unfamiliar surroundings. Most people die in healthcare facilities but are also forgotten and hidden there [39: 4].

The problem addressed here was thus related to a general critique of how societal developments have contributed to institutionalising death and dying, resulting in lonely deaths in impersonal and unfamiliar surroundings. Consequently, societal developments and death denial was given meaning in relation to, and were projected on, the physical environment of hospitals. Altogether, these concepts and understandings constituted the narrative of *Institutionalised death denial*, which we found to dominate this episode. A proposed solution to this alleged problem was that healthcare and primary care should expand in ways that would enable individuals to decide whether their end of life should be spent in their own home or in an institution. Therefore, *Dying at home* emerged as a narrative as this was presented as a solution to the problems associated with dying in hospitals. Although it was stated that little is known about where we want to die and that dying at home requires great efforts by relatives, dying at home and being surrounded by familiar people and objects was described as likely to be preferred by many. A key concept within the narrative of *Dying at home* was familiarity where relatives were constructed as contributing to familiarity. The roles and responsibilities of relatives were however presented ambivalently, positioning relatives as both a resource and a burden for professionals. Exorbitant demands, unresolved personal conflicts during the course of life, relatives not knowing their role in hospital settings, difficulties in communication between relatives and professionals, and excessive involvement in the care were the main characteristics when relatives were constructed as a burden. Yet, relatives were also viewed as a resource, which appears in *excerpt 2*:

In most cases, relatives are a natural psychological support for a dying patient. They feel connected and have an inherent need to be helpful. This spontaneous and human asset should not be underestimated. This [asset] should be used so it becomes as fruitful as possible for all parties involved. […] However, relatives should not be regarded solely as potential assistants in the bodily and human care of the sick person. Many are in a situation of crisis and are in need of care themselves [36, pp. 143–144].

Despite this contradictory portrayal, emphasis was on the relative as an asset in end-of-life care. The understanding of society as not acknowledging death and dying also motivated the need for standards and norms, which *excerpt 3* highlights:

From both the public and the healthcare perspective, care of the dying (terminal care) is in need of improvement. This is mainly because in Sweden and, until recently, the rest of the world, there has been a lack of formulated standards or defined objectives for this kind of care. The lack of such standards may partially be due to the fact that care of the dying is given such little consideration in society and in health care [36, p. 29].

What appears in the excerpt is that lack of norms and standards is explained by lack of attention to death and dying. Consequently, the narrative of *Institutionalised death denial* served as a point of departure for motivating the need of standards and norms. The source of inspiration for these norms came, according to the authors of the investigation, from the publication *Standards of Care for the Terminally Ill*. These standards were developed by a group of thanatologists, which was an emerging field of science at the time, with a focus on death and dying through interdisciplinary perspectives where a point of departure was the need to talk about death and dying in society.

Initiatives to set norms and standards were dominated by claims that end-of-life care should acknowledge the psychology of the dying person and not focus solely on the body, and hence should be conducted in a ‘psychologically correct manner’ [36, p. 101]. In the government investigation, the psychology of the dying was related to the psychological dimensions of, and reactions to, dying, which were categorised in stages that the dying person was considered to go through. These stages, dimensions and reactions were believed to follow a common pattern that staff working with dying persons should be aware of. Furthermore, psychological care was characterised by attending to universal human needs, providing open, warm and compassionate care and not letting anyone die alone. However, a person who just sits in the room and reads or whispers with others is not enough to satisfy human needs. To enable a human and personal dialog with the dying person, psychological knowledge was needed about how to conduct a warm and human dialog and how to deal with the anxiety a conversation of this type may evoke in the dying person [36, p. 122]. To facilitate this type of care, it was argued that healthcare staff should be trained and educated in psychological end-of-life care, regarding both their own reactions and the reactions of the dying person. The need for education, training and changes in attitudes was motivated by the understanding that healthcare staff were mainly educated in how to save and extend lives. This focus on psychological care deviated from the general international embrace of the hospice movements care philosophy with its emphasis on total pain, acknowledging social, emotional, physical and spiritual needs of the dying. One reason for this difference can be found in that the authors of the government investigation stated that Loma Feigenberg’s dissertation [8], focusing on the psychological reactions of terminally ill cancer patients, was of great inspiration for the investigation. Swedish policies also deviated, in an international comparison since there was a critique of the hospice movement’s care philosophy because of its strong Christian emphasis. Additionally, the establishing of specific units, a key idea of hospice care, was not considered to correspond with the Swedish ambition of equal health care for all. Moreover, the “charitable commitment” of physicians and other staff in relation to dying patients could be reduced, if they were not to be responsible for both caring for those experiencing improvement in health and those facing death [36, pp. 100–101].

### Episode 2: 1980s–1990s. Re-Domestication and Prioritisation of Dying and Death

The 1980s was a period characterised by low activity with only a few policy-documents on issues relating to end-of-life care being published. However, during the 1990s activity re-emerged, and in a government investigation on priorities in health care [37], the need for prioritising death and dying was argued for, *excerpt 4*:

How we live is important, but how we die is also important. According to the investigation, a dignified farewell from life should be one of the most prioritised rights in health care. Failing to offer dying patients’ relief from severe pain or discharging them from hospital against their will – despite there being no guarantees for the relatives or municipality to provide adequate palliative care – is unacceptable [37].

The issue of being discharged from hospitals and debates about assuming responsibility between principal authorities (municipalities or counties) were reoccurring during this episode. It was claimed that dying older people were being discharged and transferred between hospitals and nursing-homes during the final stages of life without taking their autonomy into consideration [29]. Reforms were carried out during this episode where the overall responsibility for providing service and care for older people shifted from counties to municipalities, which to a large extent shifted the place of death and dying from hospitals to nursing-homes and the own home [33]. A problem addressed within the narrative of *Dying at home* was that dying persons in need of healthcare were on the boundary between the home and hospitals, where possibilities to choose the place of death were allegedly limited*.* Furthermore, there was, focus on the concept of familiarity, which was related to the physical environment, emotions and social relations. Moreover, familiarity was associated with feeling safe and dying close to relatives [30]. Furthermore, during *Episode 2*, increased focus was on how a familiar home-like environment could be achieved through allowing personal items in a room and letting the room face landscapes and letting sunlight in [30]. Moreover, the role and needs of relatives were addressed, where it was stated that anxiety and fears among relatives could be reduced by open communication, unlimited possibilities to stay with the dying person, letting relatives participate in the care, encouraging relatives to leave the care unit at times, providing information about the formalities after death and offering relatives someone to talk to after the dying person had passed away. Also, the participation of relatives could contribute to a *natural closeness* and physical contact with the dying person [30].

Concepts stemming from the hospice movement was increasingly being used in the Swedish context, such as ‘total care’, pain relief and symptom relief [28] in contrast to *Episode 1,* which was dominated by proposals for a psychological end-of-life care and critique towards end-of-life care inspired by the hospice movement. Furthermore, during this episode, the term palliative care was increasingly used, indicating the increased breakthrough of palliative care and ambitions of ascribing certain meanings to this type of care, where ideals of the hospice movement’s philosophy at large worked as the inspiration.

### Episode 3: 2000–2018: Re-Defined and Universal Palliative Care

This episode was characterised by efforts to re-define palliative care and with ambitions of achieving universal and equal palliative care. The need to set standards was motivated by the notion that Swedish palliative care had improved but was simultaneously unequal regarding geographical distribution and access [38]. Developing common definitions would enable systematic use and was necessary in palliative care which was argued to be characterised by cooperation and work in multiprofessional teams in order to provide good palliative care [34, 38]. In a government investigation, four cornerstones of palliative care were proposed; *symptom relief, multiprofessional cooperation, communication and relations, and support to relatives* [38]. These cornerstones were based on the WHO’s principles for palliative care in combination with principles of human value formulated by the authors of the investigation. Moreover, it was argued that the palliative care philosophy, with roots in the hospice movements care philosophy, should permeate all palliative care, regardless of context and care organisation [38]. This episode was thus characterised by the narrative *Re-defined and universal palliative care*. During this episode there was an increase in publications of knowledge-support documents, care-programs, quality-indicators and evaluations aiming to achieve consensus within palliative care. In a knowledge-support document for good palliative care, several quality-indicators were formulated focusing on how well deaths were registered, times of enrolment in healthcare during the last 30 days of life, oral health, use of opioids and sedative medicine, whether symptom and pain had been estimated, occurrence of pressure ulcers, and whether there was a breaking-point dialogue (meaning that the dying person and relatives are informed about when the care changes from curative to focus on relief) [32, pp. 61–68].

Ethics was one of the key issues in this episode, where general ethical principles formulated by law in Sweden were considered applicable and of particular importance within palliative care where ethical considerations were considered to be of significance [32, p. 69]. In a care-program, the ethical platform for principles for priorities within healthcare (stating that healthcare should be prioritised in the following way: 1. human value, 2. need and solidarity, 3. cost efficiency) was defined as crucial in combination with four ethical principles; doing good, not causing harm, justice, and autonomy [34]. In a government letter [35] it was stated that quality-indicators for palliative care should rest on an ethical platform where WHO’s definitions, the principles for priorities in healthcare in Sweden and the writings from a report on advice on life-support [31] were combined. It was also noted that ethical conflicts may arise within palliative care, such as the principle of doing good, when offering treatment for symptoms which may come into conflict with the patient’s right to refuse treatment (principle of autonomy) [34].

When reflecting upon ethics, the issue of autonomy was also addressed. In [38] it was stated that an overarching aim of palliative care was that the individuals’ human value, integrity and identity should be maintained, where the ideal situation was when the person could decide for herself, as self-determination was described as a shield for integrity and if it were to fall, relatives and staff should ensure that the person’s integrity be maintained [38, p. 49]. Closely linked to these reflections on ethics were, consequently, issues of autonomy, or freedom of choice (which was the term used during this episode), regarding choice of place to die and content of care at the end of life. The lack of opportunities for freedom of choice emerged as one of the main deficiencies of palliative care. Enabling freedom of choice for patients was a crucial means of achieving good care and was suggested as a criterion for measuring the quality of palliative care in the future [38]. Furthermore, critical reflections were formulated regarding if the choice of where to die was an actual choice or if this depended on the resources and organisational structures within healthcare. Dignity was highlighted in a knowledge-support report [32, p. 23], where a general criterion for enabling quality of life and a good and dignified care consisted of symptom relief, self-determination, participation and maintaining social networks. In the report, textual sources drawn on were *The Health and Medical Services Act*, stating that human value and dignity should be respected, and a *Proposition on elder politics* emphasising that old people should finish their lives in a dignified and peaceful environment, characterised by compassion and pain relief, that they should not be transferred between hospitals and homes more than necessary, and that nobody should die alone [32]. Meanwhile, it was accentuated that the meaning of dignity is subjective. Furthermore, in this document, dignity was not given a definite meaning, since several concepts such as *human value*, *equal rights*, *the right to live* were related to dignity. The government investigation entitled *Death concerns us all – a dignified care at the end of life* [38] stated that those leaving life should receive the same love and attention as those who enter life. The conclusion made by the investigation was that a human being is first and foremost a person, regardless of whether the human being has the ability to be an actor or not.

The narrative of *Institutionalised death denial,* diminished during this episode, as the place of death and dying now, to a large extent, had transferred from hospitals to the own home or nursing-homes [38]. Consequently, the critique of how the dying were cared for in hospitals lost its significance since the solution to problems of dying at hospitals*,* now was accomplished. The narrative of *Dying at home* was, however, still present where new aspect of this narrative was the alleged lack of knowledge about the significance of the place and content of care in different locations [38, p. 34], and that dying at home could only be beneficial if relatives manage to provide care. Thus, the significant meaning given to dying at home which had been present over decades, was now met with critical reflections.

*Relatives as a unit* developed to a narrative of its own, since much focus was on the roles of relatives, where it was articulated that patients and relatives should be viewed as *a unit* [38]. Relatives were positioned as a resource and simultaneously as being equivalent to the dying person, with physical, psychological, social, and existential needs. Furthermore, it was stated that relatives should be involved in taking decisions on when the care of the dying relative should shift from life-extending care to focus on pain relief [38]. Information about the disease and dialogue based on trust between care staff, the terminally ill person and their relatives, was argued to be of importance. This trust could be achieved through continuity, openness, and honesty [34].

## Discussion

The aim of this study has been to analyse how policy narratives on palliative care have emerged and developed over time in Sweden. *Institutionalised death denial* was the narrative that served as a starting point for policy development in Sweden, from which several narratives emerged, were related to one another in varying degrees, changed in significance, and even disappeared. In particular *Episode 3* was characterised by the emergence of narratives going in new directions, where focus was on re-defining and achieving universal palliative care. Focus on quality-indicators and ambitions of achieving universal concepts could be interpreted as a general increase in bureaucratisation, medicalisation and routinisation of palliative care, illustrating a shift from the original ideals of the hospice movement, which opposed how the dying were treated in medical settings (see [3, 12, 15]). However, if the use of registers and setting standards represents this shift, it is of relevance to emphasise that wills to set standards were articulated in policies in Sweden already in the 1970s. Throughout *Episode 3*, however, in parallel with ambitions of achieving more measurable and universal palliative care, efforts were also made to elaborate and problematise ethical issues related to palliative care. Ethics, dignity, integrity, freedom of choice, person-centred care and human value were intertwined and were constructed as synonymous with good palliative care. Attention to these matters represents a re-definition of good palliative care, focusing on autonomy and dignity, which Van Brussel [21] argues represent wills to control the dying process, which is characteristic of late modern societies’ individualisation, secularisation and de-traditionalisation. The task of today, Walter argues [23], is to enable choices and attending to individual feelings, turning the individual into a consumer whom the healthcare professionals must satisfy. It is, however, noteworthy that the number of policy-documents published steadily increased over time and policies on palliative care were related to various contexts (for example, reforms of eldercare, prioritisation in healthcare and the fact that palliative care should be part of the general healthcare sector) and were increasingly specialised, through the development of indicators for palliative care and life sustaining measures. Thus, the knowledge governance of palliative care has seen a continual increase in the production of policy-documents leading to policy narratives being more diverse, specialised and fragmented over time. The tension between defining what palliative care is, but meanwhile contending its complexity has been highlighted by Khayal et al. [13], who found that the diffusion of palliative care in the UK has led to efforts to identify the ‘active ingredients’ of palliative care. Despite this, palliative care is often referred to as complex, due to its unpredictability and non-linear relations between professionals and the dying [13].

A crucial question regarding policy documents is what the purpose of these documents is, and which professions and welfare-state contexts that are addressed as receivers of these documents. This question is however not easy to answer, since professions and contexts of palliative care often were implicitly addressed. During *Episode 1,* conducting end-of-life care was a responsibility for physicians, psychiatrists and other, non-specified, healthcare staff. Moreover, society as a whole and the entire healthcare sector was made responsible, since there were claims for a need of change regarding how death and dying was viewed in society. *Episode 2* was instead marked by reforms where the well-being of older people, and therefore also the end-of-life of older people, to a large extent became, although not explicitly addressed, the responsibility of professions within eldercare. Furthermore, the entire healthcare sector was addressed since the governmental reports emphasised that death and dying needed to be prioritised within the healthcare sector. Increased emphasis on multiprofessional teams, and the importance of carrying out palliative care wherever needed made palliative care to an issue for several professions in *Episode 3.* Also, the welfare-state was designated responsibility since NBHW was given the obligation to lead the future development of palliative care in Sweden.

One key finding of this study was that the narrative *Institutionalised death denial* served as a point of departure for future developments of policies on palliative care in Sweden. Main arguments within this narrative were that healthcare staff’s orientation towards saving lives, the rapid development of medical technology, the physical environment of hospitals as an unfamiliar environment where dying persons faced a lonely death, and societal attitudes on death and dying altogether led to a construction of death and dying as being unacknowledged in Swedish society and healthcare. Through this narrative, it became possible to formulate goals with claims for standards, norms, a psychological end-of-life care and an emphasis on dying at home. A key solution to the alleged deficiencies addressed in *Institutionalised death denial* was, consequently, presented in the narrative *Dying at home*. This narrative acquired, similar to findings in studies conducted in other countries [7, 11], a central position in policies and thus became a narrative of its own, since dying at home became the norm for a good death. The meaning given to dying at home constituted what Walter [23] considers symbolising a postmodern death, with longings away from hospitals and a return to a traditional death, which was, in the policy-documents analysed, characterised by a sense of familiarity, feeling safe and being surrounded by relatives at the end of life. However, constructions of home as good places for dying and hospitals as bad places for dying lost its significance as death was argued to gradually being re-domesticated, and that palliative care should be equal in Sweden, regardless of place and that dying at home not necessarily is the prime solution to problems within palliative care. Also, it was stated that knowledge about the significance of place is limited, further contributing to reducing the divide between home as a good place and hospitals as bad places for dying.

The hospice movements care philosophy was met with critique in Swedish governmental reports during the 1970s, which stands out in an international comparison. In other countries, these care philosophies were instead embraced [5]. Despite these differences, nevertheless, the same understandings regarding death and dying in hospitals, served as the basis for the founding ideas of the hospice movement as well as Swedish policies on end-of-life care, namely a; “*symbolic critique of how dying people are managed in highly medicalised settings”* [15: 930]. However, in the subsequent episodes the understandings of the hospice movement care philosophies went from being criticised to serve as a crucial point of departure for palliative care policies in Sweden. In *Episode 2*, calls were made for holistic approaches originating from the hospice movements care philosophy to be adapted throughout the healthcare sector. In *Episode 3*, an important policy goal was that the palliative care philosophy should permeate all palliative care regardless of place and care organisation. Thus, simultaneously with processes of institutionalisation of end-of-life care in mainstream medicine under the concept of palliative care [3, 15], there were longings for the original hospice philosophy with its founding idea of ‘total care’, acknowledging physical, social, emotional and spiritual aspects of dying [6]. The tension between palliative care as an ideal of the past and a field within medicine have been highlighted by several scholars [10, 23]. Bishop [3], argues that Cicely Saunders concept of ‘total pain’ and its professional response in ‘total care’ have, through processes of knowledge production, in science, medicine and policies, in line with findings by Clark [6], developed from ideals associated with friendship and spirituality into a disciplinary power of totalitarian control, where pain among dying can be controlled [3].

## Conclusions

The results of this study highlight how policy narratives have been subject to specialisation, medicalisation, and bureaucracy through the increase of measurements, indicators, care-programs, governmental reports and evaluations. The increase of policy-documents produced by the welfare-state illustrate that death and dying have become matters of public concern through increased involvement of the welfare-state in defining what palliative care is, what problems this care context is facing and how these problems can be solved. It is however of importance to emphasise that policy narratives were located in a complex tension between medicalisation of palliative care on the one hand and the importance of acknowledging the social, psychological and emotional needs of the dying and relatives on the other. The latter was often related to the original ideas of the hospice movement’s philosophy. This tension also highlights the complexity of strives to define what palliative care is, which is often argued to be key to gaining success in the medical sphere, but simultaneously acknowledging the uncertainties and contextual differences in end-of-life care. The significant, and often non-linear, shifts in policy narratives display how notions of good palliative care, which also involves constructs of good deaths, change over time which in turn may affect palliative care in practice and the content of care that the dying person receives at the end of life.

The analysis of the policy documents has, in line with the aim of this study, provided insights on how policy narratives have developed and emerged over time. Narratives which in turn are of importance for how palliative is organised and conducted. Furthermore, this study has highlighted the development of policy narratives over time, during periods of time where palliative care developed into a field of its own in Sweden and has been subject to increased knowledge governance. Since palliative is an expanding field, more knowledge on how policy narratives develop within various contexts related to palliative care is needed. For example, it would be of relevance to highlight the intertextual relation between local, regional and national policies and differences in perspectives between the welfare-state and civil society.

## Data Availability

Authors can provide the data upon request. The data analysed in this study were public documents.

## Footnotes

1 Knowledge support documents are written to the National Board of Health and Welfare (NBHW) and serve to support to professionals within palliative care with updated knowledge.
